# A Novel Murine Model Enabling rAAV8-PC Gene Therapy for Severe Protein C Deficiency

**DOI:** 10.3390/ijms251910336

**Published:** 2024-09-26

**Authors:** Sarina Levy-Mendelovich, Einat Avishai, Benjamin J. Samelson-Jones, Rima Dardik, Tami Brutman-Barazani, Yael Nisgav, Tami Livnat, Gili Kenet

**Affiliations:** 1 National Hemophilia Center, Thrombosis & Hemostasis Institute, Sheba Medical Center, Ramat Gan 52621, Israel; einat.avishai@sheba.health.gov.il (E.A.); rima.dardik@sheba.health.gov.il (R.D.); tami.barazanibrutman@sheba.health.gov.il (T.B.-B.); ynisgav@gmail.com (Y.N.); tami.livnat@sheba.health.gov.il (T.L.); gili.kenet@sheba.health.gov.il (G.K.); 2 Amalia Biron Research Institute of Thrombosis & Hemostasis, Faculty of Medical & Health Sciences, Tel Aviv University, Tel Aviv 6997801, Israel; 3 Talpiot Medical Leadership Program, Sheba Medical Center, Ramat Gan 52621, Israel; 4 Department of Pediatrics, Perelman School of Medicine, University of Pennsylvania, Philadelphia, PA 19104, USA; samelsonjonesb@email.chop.edu; 5 Division of Hematology, Raymond G. Perelman Center for Cellular and Molecular Therapeutics, Children’s Hospital of Philadelphia, Philadelphia, PA 19104, USA

**Keywords:** protein C deficiency, gene therapy, adeno-associated virus, thrombosis

## Abstract

Severe protein C deficiency (SPCD) is a rare inherited thrombotic disease associated with high morbidity and mortality. In the current study, we established a viable murine model of SPCD, enabling preclinical gene therapy studies. By creating SPCD mice with severe hemophilia A (PROC^−/−^/F8^−^), the multi-month survival of SPCD mice enabled the exploration of recombinant adeno-associated viral vector-PC (rAAV8-PC) gene therapy (GT). rAAV8- PC (10^12^ vg/kg of AAV8-PC) was injected via the tail vein into 6–8-week-old PROC^−/−^/F8^-^ mice. Their plasma PC antigen levels (median of 714 ng/mL, range 166–2488 ng/mL) and activity (303.5 ± 59%) significantly increased to the normal range after GT compared to untreated control animals. PC’s presence in the liver after GT was also confirmed by immunofluorescence staining. Our translational research results provide the first proof of concept that an infusion of rAAV8-PC increases PC antigen and activity in mice and may contribute to future GT in SPCD. Further basic research of SPCD mice with prolonged survival due to the rebalancing of this disorder using severe hemophilia A may provide essential data regarding PC’s contribution to specific tissues’ development, local PC generation, and its regulation in inflammatory conditions.

## 1. Introduction

Protein C (PC) is a vitamin K-dependent protein synthesized in the liver. The PROC gene is located on chromosome 2 at position 2q13–14 and encodes nine exons [1]. Severe protein C deficiency (SPCD) is a rare, devastating autosomal recessive disease that presents at birth with purpura fulminans (PF) and is associated with high rates of morbidity and mortality [2,3,4,5,6,7]. As current management options are limited and burdensome, there is a large unmet need for new treatments, including gene transfer approaches. Gene therapy (GT) for hemophilia B using recombinant adeno-associated viral vectors (rAAVs) produced sufficient levels of factor IX (FIX) to prevent the need for prophylaxis; several AAV drugs have recently received regulatory approval for hemophilia B [8]. PC resembles FIX, with similar sized genes, endogenous synthesis in hepatocytes, and the requirement for vitamin K-dependent post-translational carboxylation. Further, a small increase in PC is expected to significantly ameliorate the clinical presentation of SPCD. Therefore, we hypothesize that SPCD could be treated by PC AAV GT.

Animal models are an essential research tool for the pre-clinical evaluation of GT [9]. As mice are small mammals that are easy to breed and relatively inexpensive to maintain, they are commonly used for medical research and pre-clinical testing [10,11].

While most rare bleeding disorders are compatible with life, the investigation of severe prothrombotic diseases, such as severe PC deficiency, is hampered by the limited survival of the affected mice [9]. Jalbert et al. [9] observed that mice completely lacking PC experience fatal consumptive coagulopathy during the perinatal period, resulting in a high rate of in utero death. Vetrano et al. and Lay et al. [12,13] described an alternative mouse model with a mutation leading to only 3% of normal PC levels. These mice developed severe colitis, which hindered their use for gene therapy experiments. Moreover, the residual PC levels expressed by these mice at baseline complicate the evaluation of the gene therapy intended to induce PC expression.

The concept of modulating a severe genetic prothrombotic disorder which is nearly incompatible with life by genetically combining it with severe coagulopathy may offer a valuable tool enabling the investigation and potential development of treatment modalities for this prothrombotic disorder. This is the inverse of the currently well-established observation that severe coagulopathy in mouse models can be mitigated by prothrombotic variants, such as the finding demonstrating that factor V Leiden improves in vivo hemostasis in murine hemophilia models [14].

To the best of our knowledge, no cases of SPCD combined with hemophilia have been reported in humans. However, research has explored PC inhibition as a potential treatment for hemophilia. Specifically, blocking the anticoagulant activity of APC has been shown to prevent joint bleeding in hemophilic mice, indicating that this approach holds promise for prophylactic therapy in hemophilia [15,16].

In the current study, we aimed to establish a murine model enabling the survival of SPCD mice. We then used this model to explore the potential of rAAV gene therapy for SPCD using murine PC to eliminate the possibility of a xenoprotein immune response and other cross-species differences.

## 2. Results

### 2.1. The Development of a Viable PC-Deficient Mouse Model

The breeding of heterozygous PC-deficient mice yielded only 3/200 homozygous SPCD mice instead of the 50/200 expected by Mendelian inheritance, which is indicative of its near-complete embryonic lethality. The postnatal survival of these three homozygous SPCD mice was less than 24 h, which is consistent with previous reports [9].

In order to develop a viable severely PC-deficient mouse model with undetectable plasma PC levels, we investigated the modulation of the severe thrombotic disorder of SPCD by its genetic combination with the severe coagulopathy of hemophilia A (HA): i.e., breeding heterozygous PC mice with severe HA mice. This approach yielded homozygous SPCD mice with severe HA (PROC^−/−^/F8^−^).

**Figure 1:**
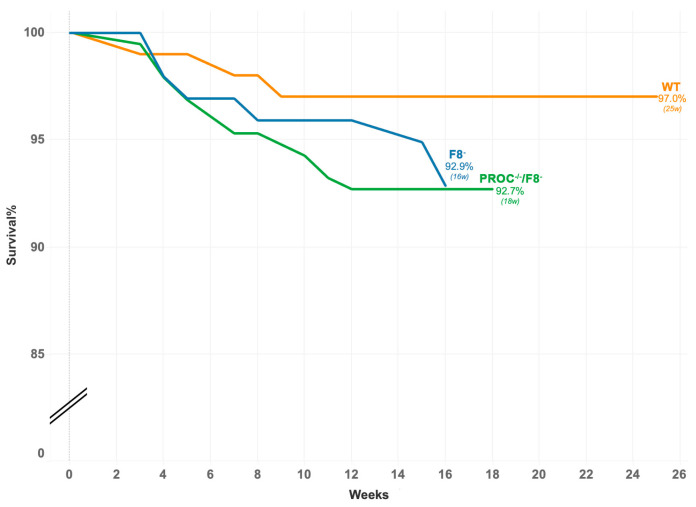
Kaplan–Meier survival curves of wild-type *(n* = 101), PROC^−/−^/F8^−^(*n* = 98), and F8^−^(*n* = 192) mice.

The WT mice (*n* = 20) and HA mice (*n* = 20) exhibited comparable PC antigen levels. In PROC^−/−^/F8^−^mice, neither PC nor FVIII antigens were detected, and mRNA extracted from the liver confirmed the absence of both PROC and F8 expressions, validating the PROC^−/−^/F8^−^murine model. A Kaplan–Meier survival analysis (Figure 1) demonstrated no significant difference in survival between the PROC^−/−^/F8^−^, WT, and F8^−^mice. The PROC^−/−^/F8^−^ mice exhibited a substantially prolonged survival compared to the homozygous SPCD mice, which all died within the first day. The postnatal survival of PROC^−/−^ mice could not be included in Figure 1 because of their limited numbers due to their near-complete in utero death.

### 2.2. rAAV8-PC GT for PROC^−/−^F8^−^ Mice

Ten 6- to 8-week-old PROC^−/−^/F8^−^ mice were administered 10^12^ vg/kg of AAV8-PC via a tail vein injection. Their PC antigen and activity levels were sequentially monitored for 12 weeks.

**Figure 2:**
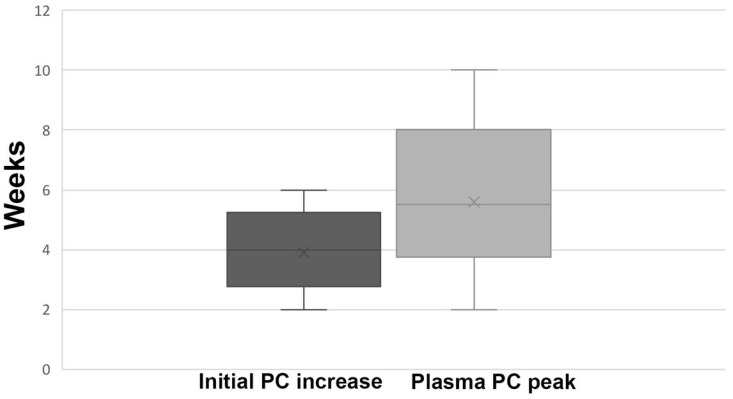
Time to increase and peak of plasma protein C antigen following gene therapy. The left bar presents the time range, in weeks, to the initial PC increase following GT. The right box presents the time range to the peak plasma PC level.

GT treated mice exhibited variable kinetics in the time until the initial detection of PC antigen in their plasma (Figure 2), though all 10 animals had measurable plasma PC levels. The initial detection of PC antigen levels occurred 2–5 weeks post vector infusion.

**Figure 3:**
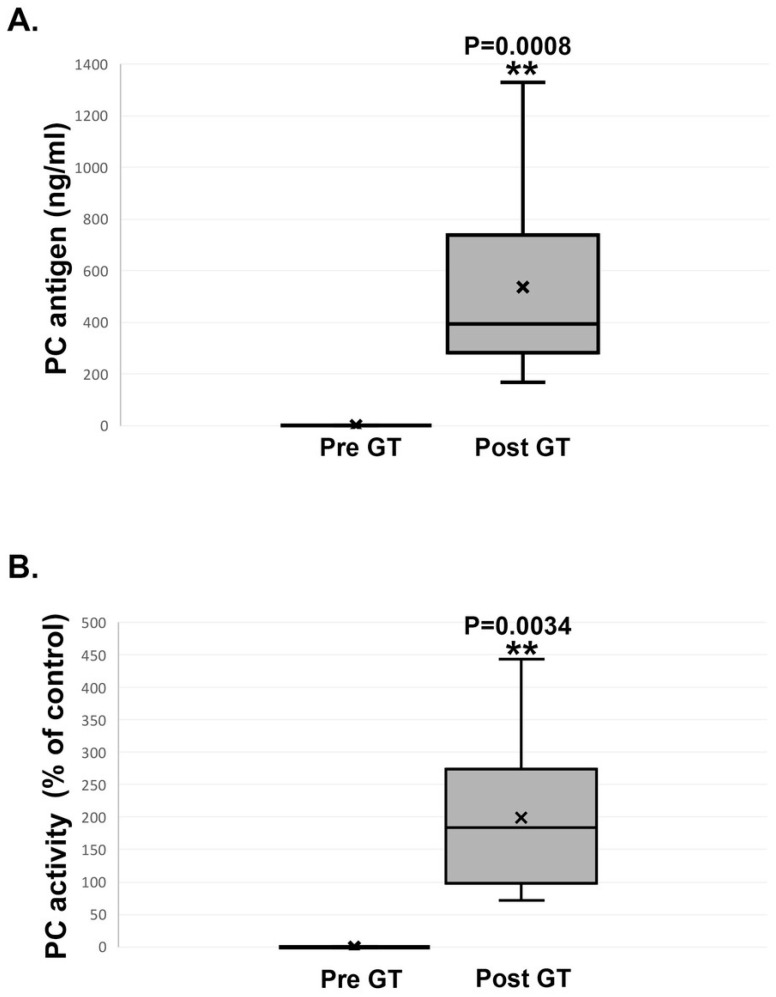
Protein C antigen and activity at baseline and at week 8 following GT. Panel (**A**) presents antigen level (ng/ml) in PROC^−/−^/F8^−^ mice before receiving GT (pre GT) and following GT, at week 6–8 (post GT). Panel (**B**) presents activated PC level (% of control) in PROC^−/−^/F8^−^ mice before receiving GT (pre GT) and following GT, at week 6–8 (post GT).

Peak plasma PC concentrations were reached at a median of 6 weeks, similar to FIX gene transfer in mice [11,17]. Individual maximal PC levels measured between 2 and 10 weeks post GT ranged from 166 to 2488 ng/mL, with a median of 714 ng/mL (Figure 3A).

After reaching peak PC levels, all mice maintained high levels of PC until at least 12 weeks after GT. As expected, PROC^−/−^/F8^−^ mice *(n* = 10) that did not receive rAAV8-PC had undetectable PC antigen levels.

In accordance with PC antigen levels, a significant increase in PC activity levels was observed following the rAAV8-PC treatment (Figure 3B).

The peak PC activity levels in these mice were 300 ± 60%, compared to 100 ± 20% in the WT control group (*p* = 0.0034) and the undetectable level in the untreated control PROC^−/−^/F8^−^ mice. Overall, these results demonstrate that rAAV8-PC administration results in the expression of functional PC at therapeutically relevant levels.

**Figure 4:**
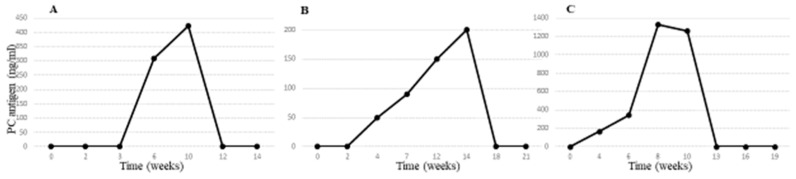
Individual responses of PC antigen following GT. Panels (**A**–**C**): (**A**) Each graph represents an individual response to PC antigen levels following GT in a different mouse. (**B**) Confirmation of PC expression in the liver following GT. (**C**) PC staining in the liver post GT.

The variable individual responses to GT are illustrated by three representative graphs in Figure 4, which show differences in the peak and total PC levels over time. All mice exhibited a variable lag time from the injection of rAAV8-PC until the initial detection of PC antigen in their murine plasma. For most mice, this lag time spanned 2–4 weeks (Figure 4A,B). However, in some mice, a more immediate increase in PC was observed. The pattern of PC increase varied among the mice: it was gradual in some (Figure 4A,B), while others experienced a sharp, steep peak (Figure 4C). After reaching peak PC levels, which yielded a wide range of plasma PC concentrations, all mice maintained high levels of PC for at least a couple of weeks. Notably, a slower decrease in PC antigen to undetectable levels was observed in some mice, with plasma PC persisting for up to 18 weeks (Figure 4B). The rate of PC decrease was not correlated with its peak level. This amount of variability in secreted transgene products is consistent with both preclinical and clinical AAV gene transfer studies [18].

**Figure 5:**
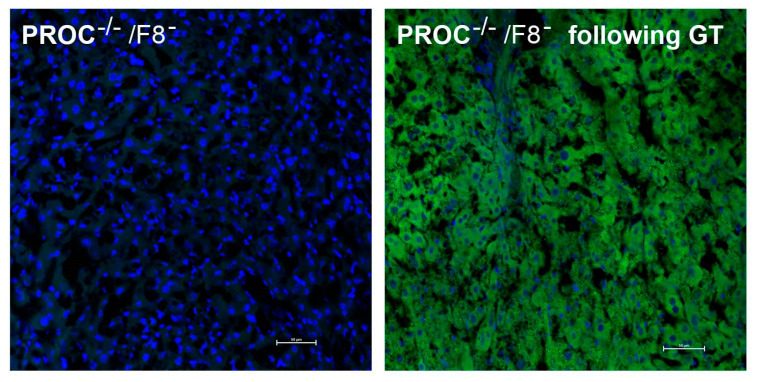
Immunofluorescence staining of protein C in the liver. Representative images compare the presence of protein C in the livers of PC-deficient mice that received gene therapy (**right** panel) to those that did not (**left** panel). Livers were stained with a protein C antibody. Significant protein C staining (green) is visible in the gene therapy-treated mice, whereas the left panel shows no green staining in PC-deficient mice. Nuclei are stained blue (DAPI). Scale bar = 50 µm; magnification = ×400.

PC’s expression in the liver following GT was confirmed by immunofluorescence staining. All mice exhibited PC staining in the liver following GT. Figure 5 illustrates PC staining in representative liver sections derived from untreated PC-deficient mice (PROC^−/−^/F8^−^) and PC-deficient mice post GT (PROC^−/−^/F8^−^ following GT). No PC protein was detected in the liver of PROC^−/−^/F8^−^ mice before GT. In contrast, pronounced PC staining was detected in the liver of the mice that underwent GT, consistent with a high plasma PC antigen level )560 ng/mL).

## 3. Discussion

In recent years, the FDA has approved six AAV gene therapies for use in the US and has received more than 900 investigational new drug (IND) applications for clinical studies. At present, more than 3400 active gene therapy trials are taking place worldwide, according to ClinicalTrials.gov [19]. Successful gene therapy approaches require extensive validation in an experimental model system prior to clinical trials. Murine models enable studies of vector efficacy, including the function of transgene products, and provide a critical system in which to investigate safety.

Appropriate animal model selection is one of the most important steps to help progress GT research into clinical trials. Previous murine models have been used for both hemophilia A [17,20] and B [11] to examine the feasibility of GT. Notably, both FVIII and FIX, which are pro-coagulant proteins produced in the liver, show a prominent similarity to PC, a natural anticoagulant, in terms of the ability of their partial restoration of protein levels to ameliorate a severe disease phenotype. That is, even a modest increase in the plasma levels of these proteins can significantly improve the clinical phenotype. Although rare, hereditary PC deficiency presents a significant health challenge with devastating consequences. Disease manifestations are described in multiple systems and include vascular, ocular, and brain abnormalities. Moreover, recent studies have broadened our understanding of the role of coagulation factors, including PC, beyond hemostasis, highlighting their involvement in inflammatory and tissue repair processes [21]. Despite the recognized importance of PC in preventing thrombosis and its catastrophic deficiency outcomes, and while the systemic pathologies of PC-deficient patients underscore its critical role, no basic data are available regarding PC’s generation and tissue-specific regulation. One major reason for the lack of data is the absence of animal models due to the poor survival of SPCD mice, which significantly hampers our ability to study the full spectrum of PC effects in vivo. The early mortality seen in animal models most probably mirrors the life-threatening complications observed in humans [3,9].

In order to overcome the major challenge of early mortality in the SPCD animal model, which precludes its use for examining GT, we present here a novel murine model of combined PC and FVIII deficiency that supports the survival of SPCD mice and enables PC gene therapy studies. PC deficiency was confirmed by DNA genotyping, as well as by the absence of PROC mRNA, PC antigen, or PC activity in plasma.

Our strategy was specifically designed to address the limitations of existing SPCD models and provide a robust framework for evaluating gene therapy as a potential treatment for this severe coagulation disorder. A mixed murine model combining SPCD and severe HA was crucial for achieving complete PC deficiency at baseline, thus enabling accurate gene therapy experiments. Consistent with previous reports [9], we observed that homozygous SPCD mice do not survive to birth due to severe thrombosis and consumptive coagulopathy, which makes it impossible to study gene therapy using this model. To overcome this limitation, we developed a mixed model by crossing SPCD mice with hemophilic mice, enabling us to generate offspring with zero PC levels. This approach was vital for testing gene therapy strategies, as it provided a stable platform to evaluate the direct effects of PC gene replacement without the confounding influences of inflammation or partial PC levels, which are present in other SPCD models [9,13]. Although the primary goal of creating this mixed mouse model was to explore gene therapy for SPCD, recent studies by Magisetty et al. and Jiang M et al. have [15,16] demonstrated that a selective inhibition of APC’s anticoagulant activity, without affecting its cytoprotective signaling, reduced the severity of hemophilic arthropathy in FVIII-deficient mice. The mixed model developed by us could be particularly useful for exploring such targeted interventions, providing a unique opportunity to understand how modifying one pathway (such as APC signaling) might mitigate the clinical manifestations of both hemophilia and SPCD. We are currently characterizing this mixed model in greater detail, focusing on its inflammation, thrombosis, and bleeding phenotypes. These assessments, alongside liver histology and inflammatory markers, are ongoing and will provide a more comprehensive understanding of the model’s utility for studying targeted interventions for coagulation disorders.

We used this viable SPCD model to determine the translational potential of the rAAV-based gene transfer of PC. We observed significant increases, up to the normal range, of both PC antigen and activity levels after gene therapy. These results provide the first proof of concept of GT for SPCD. Importantly, the safety of the rAAV vector used in this study has been extensively evaluated in human clinical trials and is FDA-approved for treating hemophilia, bolstering the potential for its application in other therapeutic areas [22].

The concept of modulating a severe genetic bleeding disorder with thrombophilia is used in clinical practice, such as anti-thrombin (AT) or PC inhibition in hemophilia [23,24], as well as in animal models such as the FV Leiden mutation in hemophilia mice [14]. Here, we present the inverse concept: a severe prothrombotic disorder, which is practically incompatible with life in mice, modulated by its genetic combination with severe coagulopathy. Such models may offer a valuable tool that enables the investigation and potential development of treatment modalities for this prothrombotic disorder.

Our study has several limitations that should be considered when extrapolating these results from murine models to humans. Firstly, PC may have a shorter half-life in mice compared to humans, similar to the phenomenon observed with FVIII.

Secondly, this is not a pure PC-deficient mouse model, as it has been combined with hemophilia to enable survival; therefore, we cannot study all the aspects of PC’s effects on plasma using this model.

As previously reported in gene therapy murine models, xenoimmunity may develop. Immunocompetent HA mice generally develop a xenoprotein immune response against human FVIII after exposures via protein administration or GT, but typically not against mouse FVIII [25,26,27]. As such, preclinical efficacy studies of non-murine FVIII gene therapy have typically utilized immunosuppression or HA mice crossed with an immunodeficient model [26,27,28]. As our mouse model is not immunocompromised, we used a murine PC construct for GT to limit such an immune response and present this proof of concept.

Although SPCD is rare in humans, it remains a devastating condition with a significant unmet need for effective treatments. Our study represents the first step toward exploring the potential of GT for SPCD in humans, which warrants further investigation.

We selected the AAV8 serotype for gene delivery based on its proven efficacy and high tropism for hepatocytes, which is particularly advantageous for liver-directed PC gene therapy applications [29]. Furthermore, AAV8 has been extensively used in preclinical studies of hemophilia gene therapy in mice, leading to significant advances that ultimately resulted in Food and Drug Administration )FDA( approval for its clinical use. The use of AAV8 in murine models of hemophilia has shown a sustained expression of clotting factors such as FVIII or FIX, providing long-term therapeutic benefits [30]. Following these successful precedents, we selected AAV8 to ensure optimal transduction efficiency, based on its well-established efficacy in gene therapy for coagulation disorders.

Our study demonstrates that gene therapy using the AAV8 vector may offer a promising alternative to current treatment options for SPCD, which are often limited by frequent dosing requirements, high costs, and risks such as antibody formation and bleeding. By achieving a sustained expression of PC, this approach could provide a more reliable and cost-effective solution, reducing the risk of life-threatening thrombosis and improving patient outcomes. Additionally, this strategy could address the global challenge of PC availability and offer a long-term treatment option that is less burdensome for both patients and healthcare systems. Our study is a proof of concept, highlighting the need for larger animal models and subsequent clinical trials.

Our proposed SPCD murine model could significantly contribute to developing treatment modalities for this prothrombotic disorder. Further investigation of SPCD mice, particularly those with prolonged survival achieved by rebalancing the disorder through severe HA, may provide insights into the role of PC in the morphological development of specific tissues and the intrinsic mechanisms that modulate local PC generation and its regulation under inflammatory conditions. Additionally, the growing demand for personalized medicine and tailored therapies highlights the opportunities for developing patient-derived animal models and implementing precision medicine approaches. This aligns with our goal to customize and enhance therapeutic strategies for complex disorders like SPCD.

## 4. Methods and Materials

### 4.1. Mice

This study was conducted with C57Bl/6 PC heterozygous mice (a kind gift from Prof. Weiler, Medical College of Wisconsin, Milwaukee, WI, USA) which have been previously described [9], severe hemophilia A (HA) mice (purchased from Jackson Laboratory, Maine, ME, USA) [29], and wild-type (WT) C57Bl/6 mice (purchased from Envigo, Nes Tziona, Israel). PC ^−/−^/F8^−^ mice were created by breeding PC ^−/+^ and HA (F8^−^) mice. All genotype-based selections were conducted by PCR, using appropriate primers for the murine PROC and F8 genes.

### Primers for Genotyping

PC:

Forward: CGTGATGAGTTTCAGGCAGTGAGAG

Reverse—wt: GCACACGTGTTGACCAGGGATAAT

Reverse Null: ACAAGCAAAACCAAATTAAGGGCCA

F8:

WT:

Forward: CCTCTTCAGGTAACTTTCAAA

Reverse: AATAAAGAACGGCTTACAAG

Mutant:

Forward (MC-18): GAGCAAATTCCTGTACTGAC

Reverse (NEO-R): TGTGTCCCGCCCCTTCCTTT

Mice were bred and maintained under specific pathogen-free conditions in the Laboratory Animal Experimental Center at Sheba Medical Center, which is affiliated with Tel Aviv University. The animal use committee at Sheba Medical Center approved these mouse studies.

### 4.2. Blood Sampling

Plasma murine samples were collected from the facial vein into 0.5 mL tubes pre-coated with 0.109M sodium citrate, with a final blood–anticoagulant ratio of 1:1 (vol/vol). After centrifugation (1000× *g* for 10 min), the plasma aliquots were stored at −80 °C.

### 4.3. Construction of Recombinant Adeno-Associated Viral Vectors

**Figure 6:**
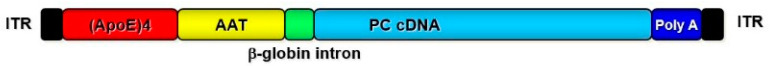
Expression cassette of murine protein C plasmid. This figure presents a schematic representation of the AAV-8 vector encoding protein C (PC). The transgene is under the control of a hepatocyte-specific promoter (alpha-1-anti-trypsin, AAT) with 4 copies of the Apolipoprotein E (ApoE) enhancer. The expression cassette consists of a short intronic sequence of the human Beta-globin gene, poly (A) sequence flanked by the inverted terminal repeat (ITR) derived from AAV serotype 2. The expression of zymogen PC derived from murine cDNA is represented.

The expression cassette contained cDNA and the PC-complementary DNA (cDNA) which appeared three copies of the liver-specific ApoE enhancer region and the liver-specific human alpha-1 anti-trypsin (hAAT) promoter, a chimeric β-globin/CMV intron, and the human growth hormone polyadenylation (hGH poly A) signal (see Figure 6).

Plasmids were grown in *Escherichia coli* Sure cells (Agilet, Santa Clara, CA, USA) and purified using the Qiagen Mega kit (Qiagen, Hilden, Germany) for the preparation of endotoxin-free DNA. Recombinant AAV vectors were produced using a triple transfection protocol in Hek293 cells using the calcium phosphate method as previously described [30], using plasmids expressing murine PC cDNA (see above), a second plasmid supplying adenovirus helper functions, and a third plasmid containing the AAV-2 rep gene and the AAV-8 cap gene (a generous gift from Prof. Walsh Lab, Mount Sinai, New York, NY, USA).

Vectors were purified using a commercial AAV purification kit (Takara, Shiga, Japan). The vector was osmotically stabilized in Hepes-buffered saline, pH 7.8; filter-sterilized; and stored at −80 °C prior to use. Vector titers were determined by quantitative commercial PCR (Takara, Shiga, Japan). rAAV8- PC (10^12^/kg Vg of AAV8-PC) was injected via the tail vein into 6–8-week-old mice, as previously described [27]. Briefly, after warming the mouse to dilate its tail veins, it was gently restrained. The tail was disinfected with 70% alcohol, and the vein was located. The needle was then inserted into the vein, and the virus was injected slowly (10^12^/kg Vg, a total volume of 50 microliters per mouse). Following the injection, the needle was removed, pressure was applied to the site, and the animal was monitored.

### 4.4. Quantification of Murine PC Antigen

Plasma samples were taken sequentially every 2 weeks. Murine PC antigen levels were measured using a commercial ELISA (LSBio, Seattle, WA, USA) based on colorimetric detection at 450 nm, according to the manufacturer’s instructions. PC antigen levels were determined by a comparison of the samples to the known standard curve supplied in the kit.

### 4.5. Quantification of Murine PC Activity

PC activity (activated protein C(APC)) was assessed in plasma using a fluorogenic substrate, as previously described [31]. Briefly, the reactions were carried out in black 96-well microplates (Greiner, Kremsmunster, Austria). The plasma was diluted (1:5) in Tris buffer (150 mM NaCl, 1 mM CaCl2, 50 mM Tris-HCl, pH 8.0) and activated using 3 mM of CaCl_2_ before the addition of the substrate. PC was activated using PROTAC. The cleavage of the fluorogenic substrate (Pyr-Pro-Arg-AMC, 20 µM, GL Biochem Shanghai Ltd., Minhang district, Shanghai, China) was measured at 37 °C every 2 min over 25 cycles (excitation at 360 ± 9 nm and emission at 465 ± 20 nm) using a microplate reader (Tecan; Infinite 200; Mannedorf, Switzerland). Its activity was calculated according to the linear increase in fluorescence intensity over time. The results are presented as percentages relative to a control.

### 4.6. PC Gene Expression in Murine Liver Prior to and after GT

Total RNA was extracted from homogenized murine livers using an Rneasy Mini Kit (Qiagen, Hilden, Germany) and reverse-transcribed into cDNA using a cDNA Reverse Transcription Kit (ThermoFisher Scientific, Vilniaus, Lithuania) according to the manufacturers’ instructions. cDNA samples were analyzed for PC RNA levels by a quantitative real-time PCR analysis using the ABI 7500 Fast device (Applied Biosystems, Waltham, MA, USA), using the following primers:

Forward: TGAATTTTCCATGTGGGAAACTGG

Reverse: CTGCCAAGGACTGTCACCCTG

Fragment size: 146 bp

### 4.7. PC Immunostaining in Murine Liver Biopsy

A total of 2 mice from each group (PC-deficient and PC-deficient following gene therapy) were used to evaluate the presence of protein C. Six weeks post treatment, the mice were sacrificed, and their livers were removed and fixed in 4% PFA for 2 h at room temperature (RT). These tissues were incubated with 30% sucrose in PBS overnight at 4 °C, embedded in OCT compound (Sakura Finetek, Tokyo, Japan) on dry ice, and kept at −80 °C. Serial sections of a 10 µm thickness were cut using a cryostat (Leica Biosystems, Nussloch, Germany). Sequential cryosections of each liver were incubated in 10 mM of citric acid for 10 min at 95 °C, blocked with 10% NDS for 1 h at RT, and then incubated with rabbit anti-protein C (1:250, Abcam, Cambridge, UK) at 4 °C overnight. The next day, the sections were incubated with Alexa Fluor 488 conjugated donkey anti-rabbit secondary antibody (1:100, Invitrogen, Waltham, MA, USA). Nuclei were counterstained with DAPI (Nucblue fixed cell stain, Molecular Probes, Carlsbad, CA, USA). Images were captured using a confocal microscope (Nikon Ax, Nikon, Tokyo, Japan) under the same conditions.

### 4.8. Statistical Analysis

Statistical analysis was performed with IBM SPSS Statistics (version 23.0; IBM Corp, Armonk, NY, USA). Continuous variables were presented as medians (range or interquartile range (IQR), as indicated). The results of PC’s activity are expressed as means ± SEM and reported as percentages relative to the control. A two-tailed Mann–Whitney test was used to compare the differences in PC activity; *p* values < 0.05 were considered significant.

## Data Availability

All relevant data supporting the key findings of this study are available within the article or from the corresponding author upon reasonable request.
